# Multidisciplinary approach to treating complex immune dysregulation disorders: an adaptive model for institutional implementation

**DOI:** 10.3389/fimmu.2025.1519955

**Published:** 2025-03-07

**Authors:** Lauren A. Henderson, Roshini S. Abraham, Aisha Ahmed, Lindsey Blount, Scott W. Canna, Natalia S. Chaimowitz, Shanmuganathan Chandrakasan, Bria Coates, James A. Connelly, Megan A. Cooper, Christine N. Duncan, Anthony French, Melissa Hazen, Michelle L. Hermiston, Brian Nolan, Anish Ray, Melissa J. Rose, Lisa Forbes Satter, Grant Schulert, Sara Kristen Sexson Tejtel, Tiphanie Vogel, Kelly Walkovich, Matt S. Zinter, Edward M. Behrens

**Affiliations:** ^1^ Division of Immunology, Boston Children’s Hospital, Boston, MA, United States; ^2^ Department of Pathology and Laboratory Medicine, Nationwide Children’s Hospital, Columbus, OH, United States; ^3^ Division of Allergy and Immunology, Ann and Robert H. Lurie Children’s Hospital of Chicago and Department of Pediatrics, Northwestern University, Chicago, IL, United States; ^4^ Division of Critical Care, Ann and Robert H. Lurie Children’s Hospital of Chicago, Chicago, IL, United States; ^5^ Department of Pediatrics, University of Pennsylvania Perelman School of Medicine and Division of Rheumatology, The Children’s Hospital of Philadelphia, Philadelphia, PA, United States; ^6^ Department of Immunology, Cook Children’s Medical Center, Fort Worth, TX, United States; ^7^ Department of Pediatrics, Aflac Cancer and Blood Disorders Center, Children’s Healthcare of Atlanta, Emory University School of Medicine, Atlanta, GA, United States; ^8^ Department of Pediatrics, Vanderbilt University Medical Center, Nashville, TN, United States; ^9^ Division of Rheumatology and Immunology, Department of Pediatrics, Washington University in St. Louis, St. Louis, MO, United States; ^10^ Pediatric Hematopoietic Cellular Therapy, Dana Farber/Boston Children’s Cancer and Blood Disorders Center and Department of Pediatrics, Harvard Medical School, Boston, MA, United States; ^11^ Division of Pediatric Rheumatology/Immunology, Department of Pediatrics, Washington University School of Medicine, St. Louis, MO, United States; ^12^ Division of Immunology and Department of Pediatrics, Division of Pediatric Hospital Medicine, Boston Children’s Hospital, Boston, MA, United States; ^13^ Department of Pediatrics, UCSF Benioff Children’s Hospital, University of California, San Francisco, San Francisco, CA, United States; ^14^ Division of Rheumatology, Ann and Robert H. Lurie Children’s Hospital of Chicago, Chicago, IL, United States; ^15^ Texas College of Osteopathic Medicine, The University of North Texas Health Science Center and Department of Hematology/Oncology, Cook Children’s Medical Center, Fort Worth, TX, United States; ^16^ Division of Pediatric Hematology and Oncology, Nationwide Children’s Hospital and Department of Pediatrics, The Ohio State University College of Medicine, Columbus, OH, United States; ^17^ Department of Pediatrics, Division of Immunology, Allergy, and Retrovirology, Baylor College of Medicine, Houston, TX, United States; ^18^ William T. Shearer Center for Human Immunobiology, Texas Children’s Hospital, Houston, TX, United States; ^19^ Division of Rheumatology, Cincinnati Children’s Hospital Medical Center and Department of Pediatrics, University of Cincinnati College of Medicine, Cincinnati, OH, United States; ^20^ Division of Pediatric Cardiology, Department of Pediatrics, Texas Children’s Hospital, Baylor College of Medicine, Houston, TX, United States; ^21^ Division of Rheumatology, Department of Pediatrics, Baylor College of Medicine, Houston, TX, United States; ^22^ Pediatric Hematology/Oncology, C.S. Mott Children’s Hospital, University of Michigan, Ann Arbor, MI, United States; ^23^ Department of Pediatrics, Division of Critical Care Medicine, UCSF School of Medicine, University of California, San Francisco, San Francisco, CA, United States

**Keywords:** multidisciplinary teams, collaborative management, immune dysregulation, institutional implementation, quality improvement research, hemophagocytic lymphohistiocytosis

## Abstract

Patients with immune dysregulation may present with varying combinations of autoimmunity, autoinflammation, immunodeficiency, atopy, lymphoproliferation, and/or malignancy, often with multisystem involvement. Recognizing specific patterns of immune dysregulation, coordinating and interpreting complex diagnostic testing, and choosing initial (often empiric) treatment can be challenging. Centers are increasingly assembling multidisciplinary teams (MDTs) to standardize evaluation and optimize treatment of patients with complex immune dysregulation (immune dysregulation MDTs [immMDTs]). However, published information on the composition and function of immMDTs is sparse, and there is little guidance for those seeking to establish or optimize an immMDT. To inform this review, we assembled a panel of 24 pediatric providers from multiple specialties who actively participate in immMDTs to provide expert opinion. We also conducted a search of the available information on pediatric immMDTs from PubMed. Based on these insights, we summarize the structure and function of active immMDTs across the United States and focus on best practices and context-dependent solutions that may enable institutions with varying goals, patient populations, and resources to establish an immMDT.

## 1 Introduction

Immune dysregulation disorders comprise a diverse group of conditions in which intrinsic immune system dysfunction causes reduced and/or amplified immune responses, driving inflammation and immunopathology (1–4). Patients with immune dysregulation may initially present with a wide spectrum of symptoms and end-organ involvement, which can make diagnosis of underlying immune dysregulation and further management difficult (1). Frequently, these patients present with unique, private, “N of 1” syndromes that do not lend themselves to guidelines, standards, or the other typical means that facilitate rapid diagnosis and treatment in more common disorders. The multiorgan presentation of many immune dysregulation disorders necessitates the involvement of a variety of specialties, which, without a collaborative management approach, can result in inconsistent treatment and incomplete care (1–4). Additionally, the rapid expansion of new phenotypes, testing options, genetic associations, and treatments presents immense opportunities for improving the care of patients with immune dysregulation. Remaining aware of these advances requires increasing degrees of specialization and cooperation.

Collaborative management of immune dysregulation with a multidisciplinary team (MDT) within a hospital has been shown to improve outcomes in patients (5–8). MDTs promote collaboration between providers from varied specialties and use of standardized algorithms and workflows to optimize care. MDTs act as a central hub for research participation, knowledge aggregation, and consensus building, which can lead to timelier, more favorable outcomes (9–11). These teams are especially important for addressing immune dysregulation, as they bring together multiple specialists to facilitate the comprehensive, sometimes highly specialized workup that is necessary for diagnosis and treatment (9–11). For this reason, immune dysregulation MDTs (immMDTs) often arise organically to address the complex needs of patients with immune dysregulation.

Despite the logic supporting their creation and recent proliferation, a comprehensive review of the various MDT models and strategies to optimize construction and function of immMDTs is not well documented in the literature. Furthermore, establishing an MDT can be complicated, and questions surrounding composition, workflow, consensus building, recruitment, financial resourcing from institutions, and sustainability are frustratingly common (9–11). This report aims to address these issues by summarizing the benefits of MDT care and describing the diverse types of immMDTs that are currently in use. Secondly, we provide a framework for the implementation of an immMDT, which can be adapted to a wide range of institutions to meet their unique needs and goals. We also provide suggested outcomes and process measures to help determine immMDT success.

Given its role in propelling the development of several immMDTs, hemophagocytic lymphohistiocytosis (HLH)/macrophage activation syndrome (MAS) will be used as the prototypical example of immune dysregulation and a model throughout this review.

## 2 Methods

This review aims to discuss the purpose, benefits, and organization of multidisciplinary care for complex immune disorders in pediatric patients, with HLH/MAS serving as a model disease, with the goal of providing an adaptable strategy for implementation. Given the limited literature and published studies on immMDTs, we convened a panel of 24 pediatric providers who actively participate in immMDTs to provide expert opinion on this topic. Members of the immMDT panel represented multiple specialties in the field of pediatric immune dysregulation: allergy, cardiology, critical care medicine, hematology, immunology, oncology, pathology/laboratory medicine, and rheumatology. The panel met during 2 in-person workshops (Chicago in June of 2022; Philadelphia in June of 2023), a hybrid virtual/in-person meeting in September of 2023, and multiple virtual meetings. Prior to the first meeting, panelists completed a structured interview to document the organization and operations of the immMDTs at their institutions. Through the subsequent meetings, the following overarching topics were discussed: 1) benefits and challenges of participating in immMDTs; 2) structure and function of immMDTs; 3) steps to implement an immMDT; 4) best practices to inform building new immMDTs; 5) measuring outcomes of immMDTs. Participants were given specific topics and questions to prepare for a round table discussion at each meeting. The meeting moderators (EB and LAH) ensured that each panelist had dedicated speaking time to address each topic.

## 3 Overview of complex immune dysregulation disorders

Immune dysregulation disorders are characterized by aberrant immune system function, which often has a genetic basis but may be further impacted by medications, other illnesses, and environment (1, 12, 13). The clinical presentation of immune dysregulation varies widely, with manifestations that may overlap with other disease states, including autoimmunity, autoinflammation, atopy, immunodeficiency, lymphoproliferation, malignancy, or a combination thereof (1–3). While this is not a comprehensive list, common signs and symptoms include frequent infections as well as early–onset, recurrent/refractory, and multi–organ system autoimmunity. Multiple cohorts of patients with immune dysregulation have highlighted the high frequency of autoimmune cytopenias, lymphoproliferation, and gastrointestinal disease in this population. However, almost any organ system can be affected, leading to diverse clinical presentations (2, 4, 13, 14). The recent proliferation of diagnostic tools that profile immune effector functions, as well as the increased accessibility of genetic testing, has led to the recognition of hundreds of unique immune dysregulation disorders. There is a rapidly growing, ever-increasing array of specific and potent therapeutics to modulate immune responses; therefore, the average clinician is finding it progressively difficult to single-handedly maintain expertise in this area. This complexity has prompted the establishment of immMDTs that successfully approach this patient population and address presentations that are rare, atypical, severe, refractory, co-occurring, or novel.

## 4 Management challenges

### 4.1 Diagnostic challenges

Patients with immune dysregulation can be extremely difficult to diagnose due to the diverse range of clinical features and symptoms that may overlap with multiple disease processes, including infections, autoimmune disease, and malignancies. Even patients within the same family who share a molecular or genetic defect can present differently; this highlights the often poor genotype-phenotype correlation in these conditions (1, 2, 15, 16). These diagnostic complexities are often compounded by the fact that many patients with immune dysregulation have severe, organ-threatening disease and some patients are critically ill and require management in the intensive care unit (ICU) (5). In these patients, rapid progression and a high fatality rate require prompt diagnosis and aggressive treatment, sometimes even before definitive diagnosis, to achieve the best outcomes (17). Other patients may present with less life-threatening symptoms and can remain undiagnosed for years, experiencing multisystem autoimmunity or other symptoms of immune dysfunction, such as lymphoproliferation, atopy, or malignancy (18, 19). In both situations, avoiding diagnostic delays is paramount; however, overlapping symptoms, lack of provider experience with rare disorders, and difficulty accessing timely laboratory evaluations are major obstacles (17, 18).

It is also essential to perform diagnostic testing prior to initiation of significant immunosuppressive therapy, as the use of empiric immunosuppression or immune-modulating therapies can further obscure diagnosis by affecting cellular and biomarker analytes. In these instances, treatment-related symptoms can mimic other conditions, and it can be challenging to determine if common complications of these therapies, such as infection, are due to disease or treatment (1–4).

### 4.2 Treatment challenges

Barriers to accurate and rapid diagnosis are not the only obstacles for patients with immune dysregulation; management options are equally complex, and despite treatment, morbidity and mortality rates remain high (4). The multiorgan presentation of immune dysregulation necessitates care from multiple clinical subspecialties (1, 2, 20). Coordinating communication among all providers and creating a streamlined management plan among disparate treatment recommendations poses a significant challenge that can delay initiation of treatment as well as increase stress on providers, patients, and families (5).

Given that multiple subspecialities are involved, maintaining strong lines of communication is essential; however, this can become extremely difficult with traditional care models. Multiple inpatient consultations are often required without a standard mechanism for collaboration. Therefore, the development of a care plan and outpatient follow-up are often the responsibility of the primary clinical team (5, 21). These challenges can cause fragmented care, recommendations that do not address the dynamic progression of the patient’s illness, and absence of the continuity (including the transition from inpatient to outpatient care) that is needed to manage long-term treatment plans (5, 22–24).

### 4.3 HLH/MAS as a model for management of immune dysregulation

HLH/MAS is a spectrum of biologically distinct, but pathologically related, disorders characterized by life-threatening hyperinflammation driven by inflammatory cytokines, T-cell activation, and macrophage activation (25–28). HLH/MAS is an ideal model within which to consider the benefits of immMDTs because its features and challenges are similar to those of other immune dysregulation disorders. It is imperative to diagnose HLH/MAS early in the disease course before irreversible organ damage develops. However, it can be challenging to recognize HLH/MAS because it typically is triggered by or recognized during the evaluation for other conditions (eg, infection, malignancy, and autoimmunity) in which inflammation is expected. The hyperinflammatory characteristics of HLH/MAS often result in multiorgan dysfunction, which necessitates the involvement of multiple subspecialists. HLH/MAS is frequently severe and progresses rapidly; therefore, providers have limited time to navigate differences in opinion on management and to create a resolution (29–31). Without treatment, the mortality rate is approximately 90% in familial HLH, with a median survival of 1 to 2 months (29). Other forms of HLH/MAS are associated with lower fatality rates; however, significant morbidity is common (30). Chemotherapy regimens with etoposide are the standard for prolonging survival in most patients diagnosed with HLH (32, 33). More recently, the treatment landscape in HLH/MAS has expanded to include non–chemotherapy-based protocols such as glucocorticoids, targeted cytokine blockers, and intracellular signaling inhibitors (34–36). Due to the diverse range of symptoms that are present in patients with HLH/MAS, management can be directed by a variety of subspecialists with varying preferred treatment approaches for HLH/MAS. This can cause inconsistent uptake and divergent treatment for patients with similar clinical presentations. Relevant expert societies continually produce guidance documents for the diagnosis and management of HLH/MAS (17, 37–43). Despite these efforts, confusion persists, practice patterns fluctuate, and there has been anecdotal evidence of poor outcomes due to misunderstanding of guidance or lack of validated guidelines (44). ImmMDTs represent a novel mechanism to promote widespread use of the most up-to-date expert guidance. In this review, we focus on the implementation and benefits of the immMDT model for patients with complex immune dysfunction.

## 5 Overview of MDTs and their utility for immune dysregulation

An MDT is a cadre of healthcare providers (HCPs) from different subspecialties whose members provide specific expertise and work collaboratively to ensure optimal delivery of care. MDTs improve clinical care through collective examination of evidence and rapid application of aggregate knowledge to make decisions, solve problems, and execute tasks more efficiently than a single provider (9). MDTs are considered the gold standard in oncology and are implemented internationally for many cancers and other complex disorders (10, 11).

Although MDTs are highly diverse, many share a similar structure and common goals of improving time to accurate diagnosis, treatment efficacy, medical team satisfaction, and patient outcomes. MDTs achieve these goals by providing evidence-based care, better continuity, improved communication, and a platform for education/research integration (10, 45). Most MDTs employ a nuclear/ancillary structure, use clear referral pathways and care algorithms, and understand key tasks needed for patient care (46). In recent years, optimization of performance measures, such as quality of care and patient satisfaction, has become increasingly important, as it can directly influence reimbursement through the Centers for Medicare and Medicaid Services (47, 48).

**Figure 1:**
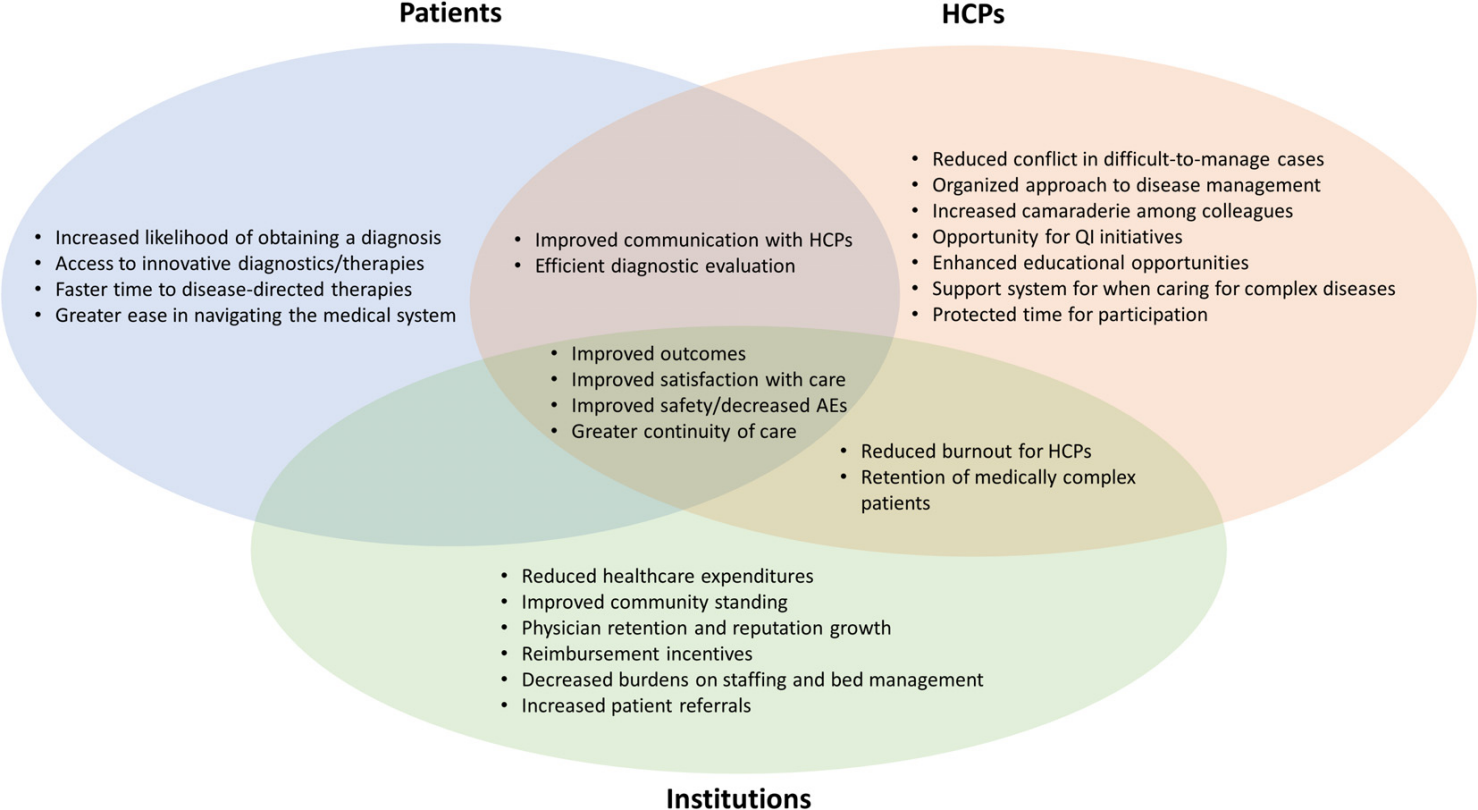
Potential benefits of MDT care for different stakeholders. HCP, healthcare provider; MDT, multidisciplinary team; QI, quality improvement.

Coordinated effort from different HCPs within an MDT improves management and produces a higher quality of care for patients with complex diseases. This can benefit patients, HCPs, and healthcare organizations (**Figure 1**) (11, 49–53). Patients experience improved safety, decreased length of hospital stay, more rapid diagnosis, greater continuity of care, consistent messaging from the medical team, and greater satisfaction with delivery of care (48–52). HCPs benefit from an organized approach to multifaceted diseases, resulting in a supportive environment for managing challenging clinical cases; this combats clinician burnout. For HCPs, MDTs facilitate streamlined communication, continuity of care, enhanced educational and research opportunities, and innovative thinking, leading to improved clinical management and professional satisfaction (50). Healthcare organizations benefit from improved efficiency, which results in reduced healthcare expenditures and decreased burden on staffing and bed management. Improved patient satisfaction can enhance community standing for a healthcare organization. MDTs can also potentially increase downstream revenue by retaining patients with complex treatment needs (47–52, 54).

Joint management across specialties has been shown to improve outcomes in patients with complex immune disorders, such as HLH/MAS, sepsis, multiple organ dysfunction syndrome, multisystem inflammatory syndrome in children, and Kawasaki disease, while increasing awareness among providers (5–8, 55–58). At one center, implementation of a standardized evidence-based guideline (EBG) for HLH/MAS within an MDT was associated with decreased mortality rates. There was also a trend toward decreased time to diagnosis, faster initiation of treatment, reduced length of hospital stays, and more rapid normalization of inflammatory markers (7, 8). The diagnostic expertise within immMDTs facilitates access to sophisticated immunologic studies and genetic testing. Several immMDTs have cited high rates of molecular diagnoses in their patient populations due to specialized testing, including identification of novel genetic causes of immune dysregulation (5, 14).

In general, immMDTs are viewed as beneficial by healthcare staff. In an anonymous survey of providers who consult in an immMDT, nearly two-thirds agreed that immMDTs improve overall patient care and are responsible for valuable changes in diagnosis and treatment of patients (5). All respondents cited good or excellent communication among members of the MDT.

### 5.1 HLH/MAS MDT models currently in use

Several types of immMDTs currently exist and can be found at academic and community institutions in the US. Some immMDTs address a single syndrome (eg, HLH/MAS), while others address all types of immune dysfunction. Some immMDTs are highly structured, while others are *ad hoc* in nature. Many have formal institutional backing, and others struggle to obtain such support; however, all face sustainability concerns. Regardless of type, all immMDTs share a similar goal of harnessing multidisciplinary expertise and streamlining quality care for patients. This goal is realized by implementing strategies to identify undiagnosed patients, reduce the time to diagnosis and treatment, increase awareness, and improve cross-specialty coordination and collaboration (5, 6, 8, 55–57).

**Figure 2:**
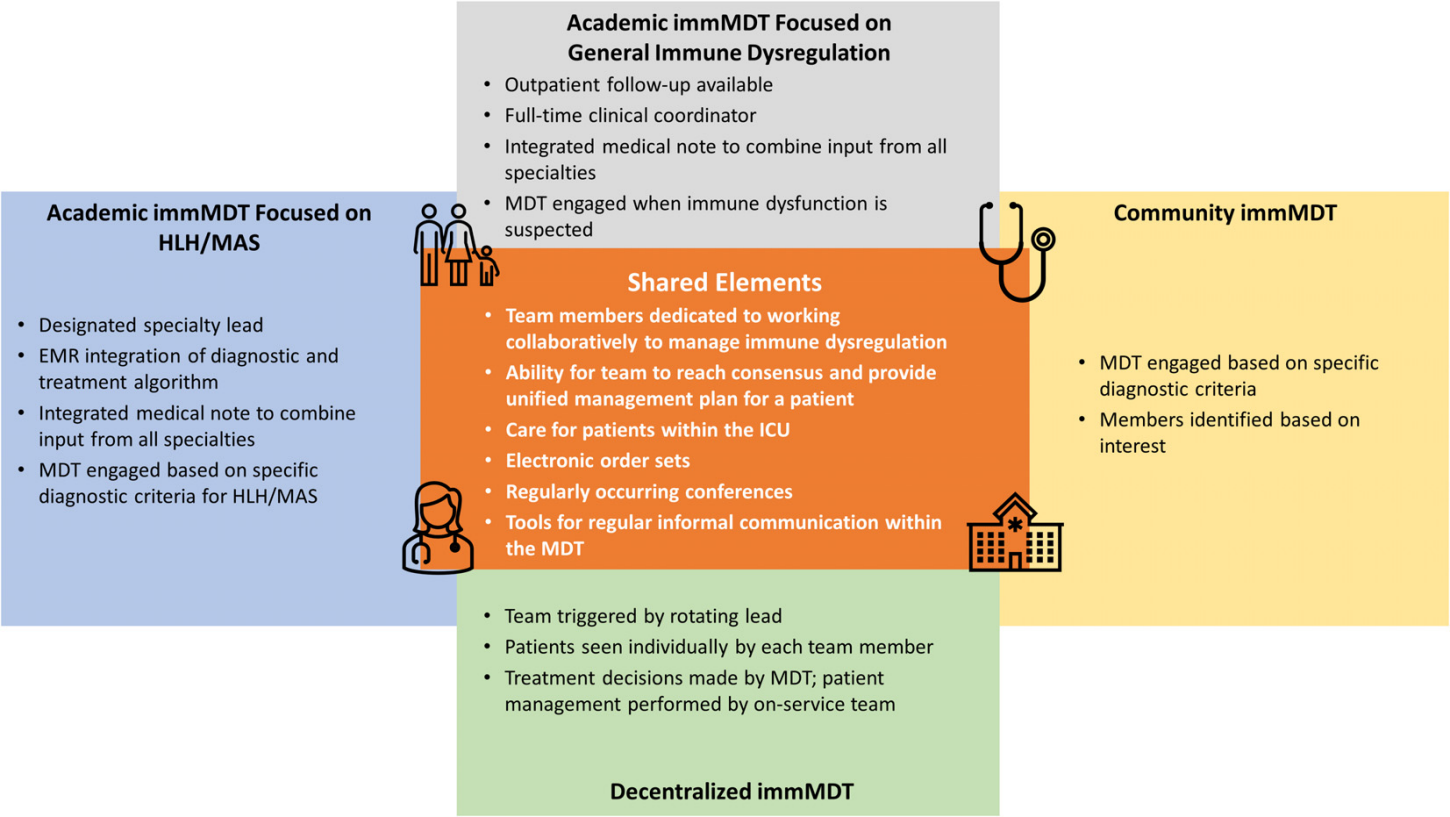
Shared elements among different immMDTs. EMR, electronic medical record; HLH, hemophagocytic lymphohistiocytosis; ICU, intensive care unit; immMDT, immune dysregulation multidisciplinary team; MAS, macrophage activation syndrome; MDT, multidisciplinary team.

Here, we summarize the various models of successful immMDTs to provide guidance for clinicians seeking to establish an immMDT at their institution (**Table 1**) (59). Illustrative examples of how immMDTs are operated, depending on the setting, are also provided (**Figure 2**) (5, 6, 8, 55–57).

**Table 1:** Key features of existing immMDTs.

	Program A	Program B	Program C	Program D	Program E	Program F
Institution Type	Academic center	Academic center	Academic center	Academic center	Nonacademic medical center	Academic center
Disease Focus	HLH/MAS	HLH/MAS	Immune dysregulation	Immune dysregulation	HLH/MAS	HLH/MAS
Dedicated Staff	None	None	APP, clinic coordinator, program manager, genetic counselor	APP	None	None
Management Tools	EBG Electronic order set for HLH/MAS	EMR tool screens PICU patients	Team provides a single, unified consult note	Team provides a single, unified consult note	Electronic order set for HLH/MAS diagnosis	Electronic order set for HLH/MAS diagnosis
Criteria to Trigger the MDT	Fever and ferritin level of ≥500 ng/mL	HLH diagnostic criteria by H score	Clinical and/or laboratory features that suggest immune dysregulation	Two of the following: ≥2 organ systems, ≥2 subspeciality teams involved, and ≥2 disease reoccurrences	Signs and symptoms that suggest HLH/MAS	Signs and symptoms that suggest HLH/MAS
Organizational Features	One specialty to direct care and determine if the patient should follow the EBG	Electronic alert when HLH diagnostic criteria are met	APP and MDT physician respond within 24 hours of referral to MDT	APP is initial point of contact and establishes which specialists are needed	HLH diagnostic criteria evaluated in patients referred to the program	Patient seen by each team member MDT makes treatment decisions together
Internal Communication	Email distribution list	Email distribution list	Rapid-response email distribution list Weekly conferences	Care discussions for each new patient within 48 hours Monthly patient review conferences	Meetings every 3 months Internal email newsletters	Email distribution list and *ad hoc* meetings
Participants	Bone marrow transplant, critical care, hematology, immunology, infectious disease, neurology, oncology, and rheumatology	Critical care, hematology, immunology, infectious disease, laboratory medicine, oncology, pathology, and rheumatology	Critical care, genetics, GI, hematology, hepatology, immunology, infectious disease, neurology, oncology, pathology/laboratory medicine, pharmacy, pulmonology, rheumatology	Critical care, hepatology, immunology, infectious disease, nephrology, oncology, and rheumatology; additional specialists (eg, a genetic specialist) may join as needed	Critical care, hematology, hospital medicine, immunology, infectious disease, neonatology, oncology, pediatrics, pharmacy, and rheumatology	Critical care, hematology/oncology, rheumatology/immunology, infectious disease, neurology, and others based on clinical need
Additional Features	Outpatient follow-up available	Specific to patients in the ICU	Outpatient follow-up available	None	None	None
Teaching	Trainee participation in consults Outreach to house staff at conferences	Multidisciplinary case review conference, which includes fellows	Medical student, resident, and fellow clinical rotations available Fellows participate in conferences	Consults, which occasionally feature in-house staff conferences	Rotating medical students participate in consults and conferences	Trainee participation in consults
Research/QI	Retrospective review of outcomes for QI metrics Patients enrolled in biobanking protocol	HLH patient research registry	Patients enrolled in biobanking protocol Externally funded research projects on specific disease areas	Biobank protocol in development	Retrospective review to evaluate order set use	None

APP, advanced practice provider; EBG, evidence-based guideline; EMR, electronic medical record; GI, gastroenterology; HLH, hemophagocytic lymphohistiocytosis; ICU, intensive care unit; immMDT, immune dysregulation multidisciplinary team; MAS, macrophage activation syndrome; MDT, multidisciplinary team; PICU, pediatric intensive care unit; QI, quality improvement.

### 5.2 Inpatient immMDTs at academic centers that treat patients with HLH/MAS

Program A uses an EBG to treat patients with HLH/MAS (6). An EBG is a clinical algorithm designed to achieve consensus on the optimal ways to manage a given condition. In program A, the patient follows the EBG when there is concern for HLH/MAS; broad criteria (fever and ferritin level ≥500 ng/mL) are used and designed to capture as many patients as possible. A key aspect of program A is that a single specialty (in this case, rheumatology) is the initial point of contact in standardized patient referral patterns. Based on clinical judgement, this service engages the HLH/MAS immMDT team and guides the diagnostic evaluation and management decisions. An order set within the electronic medical record (EMR) augments standardized diagnostic testing and medication administration (6). In addition, an email distribution list facilitates real-time input and fosters collaboration among members. A consensus must be achieved before recommendations are given to the primary team.

Like program A, program B is also at an academic center and focuses on patients with HLH/MAS (8). However, program B is unique in that it uses a taskforce to assist in the diagnosis of patients in the pediatric ICU (8). In program B, a best practice advisory that uses the HLH diagnostic criteria actively screens all pediatric ICU patients and uses EMRs to alert providers to initiate further diagnostic testing. Additionally, an EPIC tool was built to calculate an H-score to estimate risk of a hemophagocytic syndrome. Like program A, program B also uses an email distribution list to facilitate collaboration and case discussion.

### 5.3 Inpatient immMDTs at academic centers that manage immune dysregulation

Program C is at an academic center and manages all patients with immune dysregulation (55). Inpatients are referred to program C when immune dysregulation is suspected based on clinical and/or laboratory features that prompt concern from the primary team, and a consultation to the MDT service is subsequently arranged. The on-call MDT member, a dedicated advanced practice provider (APP), and responding specialist complete the consultation within 24 hours. The entire MDT team is contacted through a rapid-response email distribution list so that additional multidisciplinary input can be provided. In addition, the entire team reviews the patient’s chart during a weekly conference and weighs the benefits and limitations of different therapeutic approaches. Outpatient initial consultation or follow-up through this immMDT is also available.

Program D is similar to program C in that it is also housed at an academic center, manages immune dysregulation, and has a dedicated APP on staff (5). In program D, the APP is the initial point of contact for patients who enter the program and acts as a coordinator for the entire team. This program uses consultation criteria (≥2 of the following: ≥2 organ systems involved, ≥2 subspecialty teams consulted, and/or disease that reoccurs ≥2 times) to identify patients with immune dysregulation and multiple organ involvement. When the criteria are met, the APP takes a detailed patient history and establishes which specialists are needed. A care discussion is organized within 24 to 48 hours to gather input from relevant providers. Additionally, the immMDT holds a monthly patient review conference.

### 5.4 Inpatient immMDTs at community centers that evaluate patients with HLH/MAS

Community institutions can lack the same access to specialized resources as academic institutions; therefore, creating an immMDT can be more challenging. One such motivated institution, Program E, enlists a work group that consults on cases presenting with fever of unknown origin to improve the diagnosis and management of HLH (58). The MDT has created an electronic order set specific to the HLH diagnostic criteria for patients presenting with signs and symptoms of HLH/MAS. The work group meets every 3 months to discuss research and cases that are relevant to the diagnosis and management of HLH/MAS. This work group’s popularity grew through an internal email newsletter that includes basic disease information and the latest research in HLH/MAS. Specialties in this immMDT include hematology/oncology, infectious disease, immunology, rheumatology, neonatology, and pediatric critical care. Providers from hospitalist groups, clinical pharmacists, and rotating medical students are also included (56–58).

### 5.5 Decentralized inpatient immMDT that treats patients with HLH/MAS

Program F is a decentralized immMDT that specifically treats patients with HLH/MAS. Program F employs a rotating lead, who is a provider on service, to trigger engagement of the immMDT, and each team member sees a patient individually as part of consultations. Once a treatment decision is made by the immMDT, the on-service team continues to treat the patient. This type of immMDT preserves the autonomy of each specialist and has no centralized or administrative support.

It is important to note that programs A to F are only a few examples of immMDTs that are used to care for patients with immune dysregulation. We aimed to highlight the diverse strategies used to resolve challenges in the care of patients with immune dysregulation.

## 6 Playbook for the institutional implementation of an immMDT

**Figure 3:**
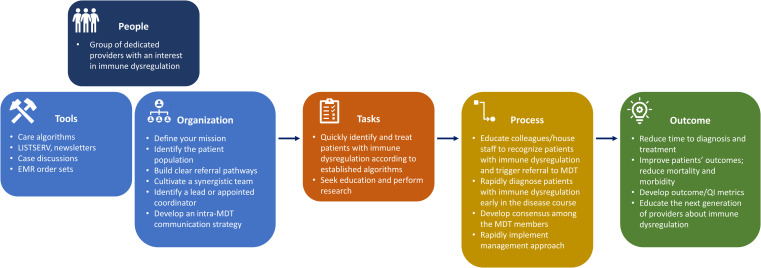
Descriptive model of the typical immMDT. EMR, electronic medical record; immMDT, immune dysregulation multidisciplinary team; MDT, multidisciplinary team; QI, quality improvement.

**Figure 4:**
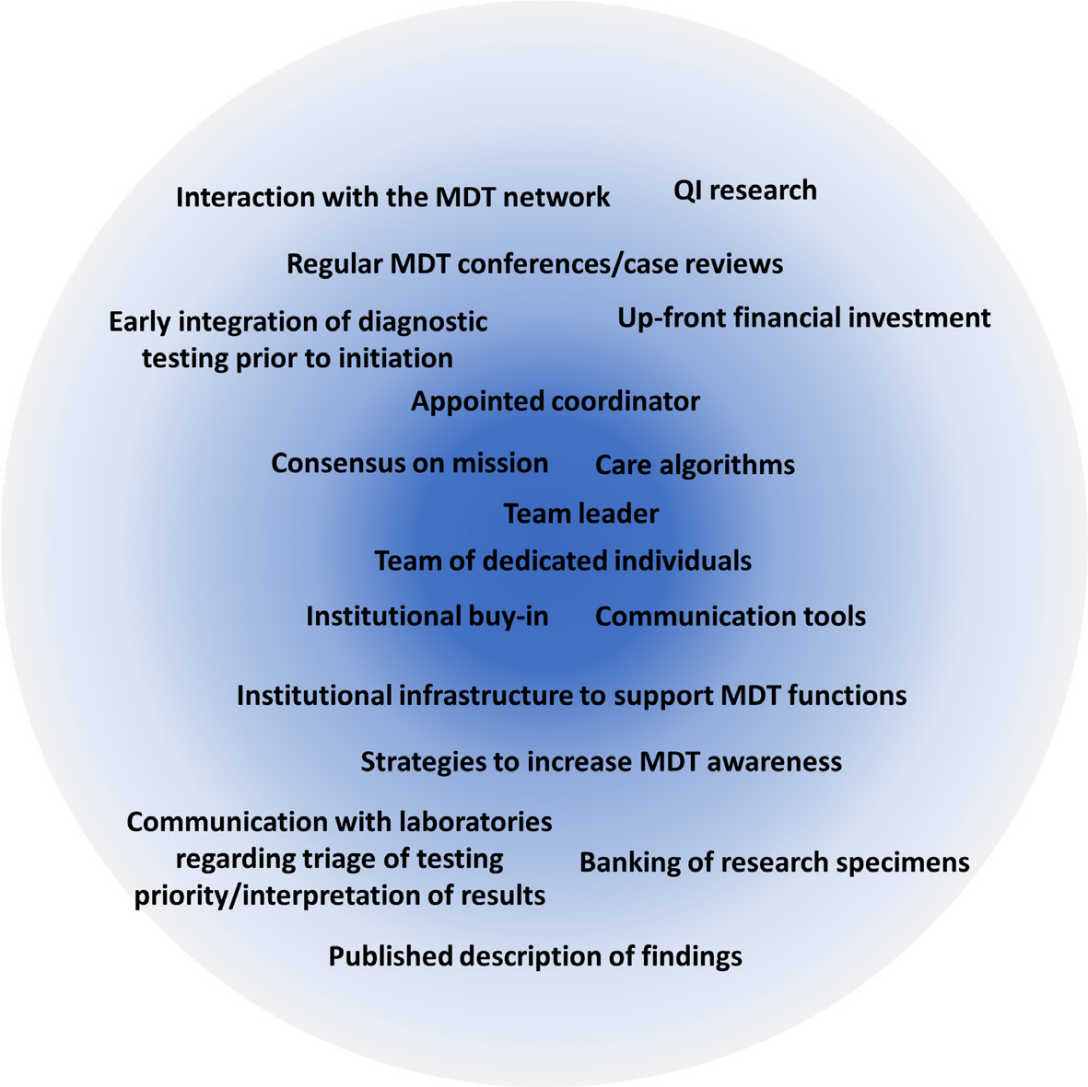
Core aspects needed to establish a context-dependent immMDT. The most essential features required for a successful immMDT are listed in the center of the circle. immMDT, immune dysregulation multidisciplinary team; MDT, multidisciplinary team; QI, quality improvement.

With careful comparison and expert discussion, we have created a guide to assist in the development and management of an immMDT (**Figure 3**). Using this framework, we believe that it is possible for institutions to build an immMDT that adapts to their available resources and the specific needs of their patient population (50). In this section, we recommend best practices for the successful implementation of an immMDT and outline aspects that are essential across all settings and those that are desirable but not necessarily required or applicable (**Figure 4**).

### 6.1 Essential components and recommended best practices

The top priority for all immMDTs should be composition and ability to establish a team across disciplines/roles. An appointed individual or dedicated staff member who can coordinate the team, manage triage/intake, and determine key players on a case-by-case basis is vital. The team should comprise a committed, stable group of experts who are supported by their division and have the capacity to participate. The group often includes physicians and advanced practice providers in immunology, rheumatology, hematology/oncology, infectious disease, critical care, and pathology/laboratory medicine, but this may vary among institutions. Subspecialists in cardiology, gastroenterology, genetics, hematology, nephrology, neurology, and pulmonology and others who frequently care for patients with immune dysregulation may also be involved. Access to bedside nurses, pharmacists, and patient advocates is highly valued. Access to social workers is also important to support the clinicians and family members caring for seriously ill children.

Once the team is created, the goal of the mission and logistics of operating the immMDT should be established. The patient population that will be served and clinical criteria that prompt engagement of the immMDT should be identified. If MDT members have differing opinions on the management of a condition, effort should be made to gain consensus and agree on an algorithm. There should be dedicated infrastructure to support team functions and tools to implement the immMDT (eg, EBGs, best practice advisories, and electronic order sets). Strategies for internal communication (eg, email distribution lists, secure internal messaging apps [Slack, Microsoft Teams], and regularly scheduled meetings) and external communications (ie, to get referrals) should also be agreed on. Dissemination of information about the MDT through the use of newsletters, flyers, notifications, and educational seminars raises awareness of the immMDT.

The acquisition of support is necessary, which should ideally come from the institution leadership and from one or more internal “champions” who would like to lead the initiative. Up-front financial investment is typically required to support the immMDT infrastructure and overall functions. A plan for billing revenue and the eventual distribution of funds is also equally important.

### 6.2 Desirable components

Once the essential elements of an immMDT have been established, additional components can be incorporated depending on the institution, patient population, and overall goals. Many MDTs choose to use regularly scheduled conferences and case reviews for communication. This type of dedicated time for communication is useful but may not be necessary or even possible in all situations. Some teams rely on algorithms and/or artificial intelligence in the EMR to trigger consultations based on clinical or laboratory criteria. While EMR automation can be beneficial, it may be most valuable to institutions that focus on a single disease rather than those that serve patients with general immune dysregulation.

Given the challenges associated with timely and accurate diagnosis of patients with immune dysregulation and because diagnosis can be obfuscated by immunosuppressive therapy, early integration of diagnostic testing prior to initiation of such therapy may be desirable for some immMDTs. Ascertaining which diagnostic test to perform and when to send it forms a cornerstone of early diagnosis and proper treatment of patients with immune dysregulation. In many centers, diagnostic immunology evaluation is often an afterthought due to the lack of awareness; thus, interpretation of results is fraught with challenges that reduce efficacy of care and treatment.

Additionally, payer support specialists and/or case managers who help facilitate financial coverage of potentially high-priced outpatient medications may be useful in some situations. Quality improvement (QI) metrics that measure outcomes may help generate institutional support and sustainability; however, these metrics can be difficult to follow and are not required to run a successful immMDT.

A focus on clinical research initiatives, including collaboration with a clinical laboratory for testing and research, is also important but not necessary. Involvement with research networks such as the United States Immunodeficiency Network, North American Immuno-Hematology Clinical Education and Research Consortium, Primary Immune Deficiency Treatment Consortium, Undiagnosed Diseases Network, and North American Consortium for Histiocytosis, among others, can be beneficial for gaining support, increasing collaboration, and generating new insights on many of these rare diseases. Association with these networks can also provide funding and educational opportunities.

## 7 Barriers to implementation of MDTs and best practices for initiation

The main barriers to implementing an MDT and expert opinions on how to overcome these barriers are summarized in **Table 2** (11, 51, 52, 60, 61). At the physician level, lack of experience and excessive workload can hinder the formation of MDTs. MDTs require dedicated time for setup and operation that may be difficult to integrate into an already full schedule. Additionally, uncertainty surrounding team building, workflow processes, billing, and overall structure can pose challenges. At the institutional level, MDTs can be inhibited by lack of support or buy-in from leadership, insufficient resources, and inadequate infrastructure. Other significant barriers to MDT formation include the need for strong internal leadership and modifying the perception that MDTs are too large and difficult for smaller institutions to replicate.

**Table 2:** Key barriers to the implementation of MDTs and proposed strategies to overcome these challenges.

Barrier	Strategy
Provider related	
Lack of experience in establishing and maintaining an MDT	• Find a motivated leader to recruit participants • Provide educational opportunities within MDTs to increase training of the next generation of providers • Leverage the immMDT network and published guidelines
Lack of time to establish and run an MDT	• Ensure dedicated effort toward MDT involvement • Use/develop implementation tools (eg, order sets) to save time • Ensure built-in time to review cases and achieve agreement in settings such as scheduled case conferences • Establish algorithms to reduce workloads/time deciding on course of action
Provider burnout	• Establish clear and well-defined protocols on the structure, frequency, and duration of meetings • Grant full-time equivalent status to the individual leading the team • Ensure a shared mental load when providing care to complex patients • Increase opportunities for professional development • Recruit advanced practice providers to facilitate the program
Providers unable to agree on management	• Standardize communication expectations surrounding complex patients to allow for questions and disagreements to be addressed by all involved providers and consensus to be achieved • Use tools such as consensus conferences to establish agreement • Encourage frequent communication among team members to ensure that trust is built
Institution related	
Lack of support/buy-in	• Increase MDT awareness early in planning through newsletters, emails, and presentations • Recognize publications and societies supporting MDTs as optimal for patient care • Establish the importance of MDTs in establishing the reputation of the institution
Inadequate infrastructure	• Implement disease-specific rather than comprehensive immMDTs • Use context-dependent solutions • Leverage the immMDT network for collective resources • Leverage QI resources for MDTs • Employ video conferencing and telemedicine to connect MDT members and obtain high-level expertise
Insufficient financial support/funding	• Obtain grants to incorporate research studies into the MDT design • Garner support from philanthropic organizations or patient advocacy groups • Track the institution’s downstream revenue that has been generated by MDTs • Highlight improved efficiency, which lessens costs for the institution • Highlight increased patient referrals due to existence of the MDTs

immMDT, immune dysregulation multidisciplinary team; MDT, multidisciplinary team; QI, quality improvement.

MDTs require strong leadership from respected experts to be successful. A highly motived leader or leaders who can ensure the active participation and collaboration of all team members are beneficial (11, 60). Due to the resource-intensive effort required, this challenge may be overcome by granting full-time equivalent status to the individual leading the team. Establishing clear protocols on the structure, frequency, and duration of case reviews can mitigate fears regarding time constraints and competing responsibilities (51). Additionally, MDTs have been shown to reduce provider burnout by fostering a sense of personal accomplishment through professional growth, skill development, and encouragement from colleagues (24).

MDTs can face institutional pushback due to disruption of the established model of care and because perspectives on certain therapies may differ between specialties (61). To gain support, strategies to increase awareness and participation may be useful. Such initiatives include garnering endorsement from institutional leadership, meeting with internal stakeholders early in the planning to align on clinical vision and overall goals, and using tools such as consensus conferences to establish agreement.

An upfront financial investment is usually necessary to establish an MDT. The actual cost will vary greatly depending on the nature of the MDT and the approach to implementation. Factors to consider in terms of cost include administrative time for the participating clinicians as well as potential opportunity costs for clinicians who may generate greater revenue in more traditional, high-volume clinics. Salary support may be needed for advanced practice providers and clinical coordinators to ensure the MDT operates efficiently. There may be costs associated with clinical space, start-up expenses, and research/QI efforts. These costs may partially be offset through improved efficiency of care and the ability to retain and treat more medically complex patients. Hospital administrations should additionally be made aware of the downstream revenues that immMDTs generate beyond direct billable events in assessing the true return on investment in supporting these teams. Additionally, to mitigate funding challenges, support from grants can be obtained from research organizations, philanthropic foundations, or patient advocacy groups (6, 7). At least 1 existing immMDT receives research funding for incorporating QI studies into its design. This not only provides financial backing but also valuable, evidence-based information on outcomes (6, 7). Support may also be obtained from patient advocacy foundations.

Interdisciplinary telemedicine meetings provide a valuable opportunity to attain high-level expertise at institutions with limited access to specialist care (60, 62). Video conferencing is cost–effective and can be used to discuss cases and improve meeting attendance by removing geographic transportation barriers (11, 51, 61). Use of the immMDT Network, which is currently under development, may also offer opportunities for collaboration, collective resources, and expert guidance for newly established MDTs.

Overall, the effective implementation of this model of care requires the active involvement of all stakeholders and development of institutional and context-based solutions that are appropriate for a particular institution and its patient population. For example, disease-specific immMDTs, rather than teams that focus on all aspects of immune dysregulation, may be easier to reproduce; they are generally smaller and easier to manage due to their narrower scope.

## 8 Measuring outcomes: Is my MDT working?

A systematic approach for measuring the effectiveness of an MDT is important for establishing the overall impact of the MDT and can be beneficial for obtaining support and/or funding; however, it can be difficult to make meaningful comparisons because there is typically no active control group, and it can take considerable time to track outcomes (45). One approach to address these challenges is to identify specific metrics to measure before and after implementation of the MDT. As more centers develop MDTs, comparisons across institutions that utilize different models for MDTs may become possible. Useful ways to initially evaluate effectiveness include measuring the number of cases discussed and determining order set use, changes in treatment plan, turnaround time for laboratory studies, amount of redundant testing, and number of research initiatives (58, 61). Ideally, programs should address the relatively simple and achievable goals of assembling a team and communicating effectively before addressing the larger objective of changing patient outcomes. Once these initial goals are achieved, metrics for clinical and patient-facing outcomes, patient/physician perspectives, administrative burden, and cost-effectiveness can be investigated (45). Observational assessment tools highlight areas in which the MDT is succeeding and those that need improvement (10).

In the context of immune dysregulation disorders, it may be useful to measure time to diagnosis, time to initiation of therapy, duration of illness (eg, fever days, length of hospital stay, and readmission rate), severity of illness (eg, organ dysfunction scores, need for ICU care, mortality, and normalization of disease-associated biomarkers), and measures of quality of life (eg, patient-reported outcomes) to evaluate the effectiveness of the MDT (6, 7, 56–58). Metrics that include an evaluation of the patient experience are important to capture, such as satisfaction with coordination of care through the immMDT, perceived timeliness of management decisions, and patient-rated outcomes. Similarly, assessing the perceptions of participating clinicians is also central to understanding the benefits of an immMDT. These QI metrics reflect the initial goal of the immMDT: to improve outcomes in patients with immune dysregulation by increasing the efficiency of diagnosis and treatment.

## 9 Discussion

Accurately diagnosing, treating, and monitoring patients with immune dysregulation is a significant challenge that can be addressed by coordinated care through an immMDT. ImmMDTs likely improve outcomes by reducing time to diagnosis, time to treatment initiation, and mortality rates in patients with complex immune dysregulation; however, more published evidence supporting this model of care is needed. Many types of immMDTs are currently in use at institutions across the US, from highly structured MDTs with dedicated coordinators to decentralized MDTs that rely on informal consensus; however, all streamline care and attempt to improve outcomes. While there is no single perfect model, there are context-dependent solutions that can be adapted to a variety of situations and goals. Here, we propose methods for the implementation of an immMDT in a variety of settings; however, the components should be tailored to the participating providers and desired patient population. The first and most important step in establishing an immMDT is to bring together a broad group of dedicated stakeholders to design and implement a multidisciplinary approach to the treatment of immune dysregulation. Clear goals and objectives and institutional support that is commensurate with those goals are necessary for long-term success and sustainability.

As knowledge and the availability of immune function testing and genetic testing increase, the patient population with immune dysregulation disorders will continue to grow and require increasingly complex multidisciplinary care. These disorders are rare, but due to complexity of care, they require extensive clinical resources from the time of diagnosis to initiation and maintenance of therapy. MDTs are hubs of knowledge and provide continuity for this complex group of patients.
